# 2-Thiohydantoin Moiety as a Novel Acceptor/Anchoring Group of Photosensitizers for Dye-Sensitized Solar Cells

**DOI:** 10.3390/ma13092065

**Published:** 2020-04-30

**Authors:** Aleksandra Bartkowiak, Bartosz Orwat, Maciej Zalas, Przemyslaw Ledwon, Ireneusz Kownacki, Waldemar Tejchman

**Affiliations:** 1 Faculty of Chemistry, Adam Mickiewicz University in Poznań, 8 Uniwersytetu Poznańskiego St., 61-614 Poznań, Poland; aleksandra.bartkowiak@amu.edu.pl (A.B.); maciej.zalas@amu.edu.pl (M.Z.); irekk@o365.amu.edu.pl (I.K.); 2 Center for Advanced Technology, 10 Uniwersytetu Poznańskiego St., 61-614 Poznań, Poland; 3 Faculty of Chemistry, Silesian University of Technology, 9 Marcina Strzody St., 44-100 Gliwice, Poland; przemyslaw.ledwon@polsl.pl; 4 Institute of Biology, Pedagogical University of Cracow, 2 Podchorążych St., 30-084 Kraków, Poland; waldek.tejchman@gmail.com

**Keywords:** thiohydantoin, DSSC, anchor, acceptor, photovoltaics, dye, TiO_2_

## Abstract

Very recently, we have reported the synthesis and evaluation of biological properties of new merocyanine dyes composed of triphenylamine moiety, π-aromatic spacer, and rhodanine/2-thiohydantoin-based moiety. Interestingly, 2-thiohydantoin has never been studied before as an electron-accepting/anchoring group for the dye-sensitized solar cells (DSSCs). In the presented study, we examined the applicability of 2-thiohydantoin, an analog of rhodanine, in DSSC technology. The research included theoretical calculations, electrochemical measurements, optical characterization, and tests of the solar cells. As a result, we proved that 2-thiohydantoin might be considered as an acceptor/anchoring group since all the compounds examined in this study were active. The most efficient device showed power conversion efficiency of 2.59%, which is a promising value for molecules of such a simple structure. It was found that the cells’ performances were mainly attributed to the dye loading and the ICT molecular absorption coefficients, both affected by the differences in the chemical structure of the dyes. Moreover, the effect of the aromatic spacer size and the introduction of carboxymethyl co-anchoring group on photovoltaic properties was observed and discussed.

## 1. Introduction

**Figure 1:**
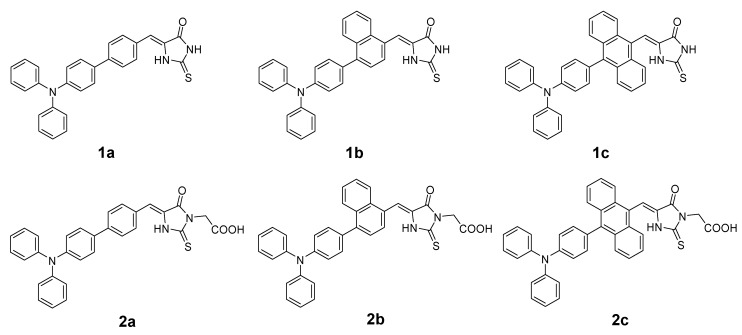
Chemical structures of the examined compounds.

Over the last two decades, the dye-sensitized solar cells (DSSCs) have gained much attention as an alternative source of energy [1,2]. Along with the development of solar energy conversion devices, the DSSC’s application concepts have evolved, and now these cells are considered as a power supply for portable electronics, the Internet of Things (IoT) devices under ambient light illumination, and as coverage for greenhouses allowing recovery of the energy from light spectrum not useful for the plants [3,4,5,6,7]. Such applications require low production costs, convenient device manufacturing methods, high efficiency, and control over the range of wavelengths being absorbed. A potential group of compounds that meet these requirements are metal-free organic sensitizers. Actually, sensitizers are the most commonly optimized elements of DSSCs because they play a crucial role in light-harvesting and photoconversion efficiency of the device [8]. In general, sensitizers contain donor (D) and acceptor (A) moieties between which electron transfer occurs as a result of light absorption. On the molecular level, absorption of light involves the electron transfer from the highest occupied molecular orbital (HOMO) to the lowest unoccupied molecular orbital (LUMO). To convert photoexcitation into photocurrent, the LUMO energy should be higher than that of the anode Fermi level. Moreover, the part of the dye in which LUMO is located must be in close proximity to the anode surface to facilitate electron injection. Therefore, it is highly desirable to improve the binding of the acceptor part, where LUMO is located, to the electrode surface. In view of the above, the acceptor part is often assumed to act as an anchor. The affinity of the anchor to the electrode determines the electrode’s surface coverage, molecule’s orientation, charge injection, and photostability, which highlights the key role of the anchor. Up till now, carboxyl, pyridyl, phosphonate, cyanate, rhodanine-based, and many other anchoring moieties have been proposed [9,10,11,12,13,14,15,16,17,18]. Particularly interesting are the derivatives of rhodanine since they have many heteroatoms in their structure, which can simultaneously act as binding sites. In fact, sensitizers equipped with this moiety have been extensively studied and proved to be quite efficient [19]. Recently, we have synthesized and evaluated biological properties of a series of new merocyanine dyes composed of triphenylamine, π-spacer, and 2-thiohydantoin moieties, the latter being an analog of rhodanine in which the sulfur atom in position 1 is replaced by an –NH– group [20]. The structures of the above-mentioned compounds consist of an electron donor bonded to an electron acceptor through a π-aromatic spacer (Figure 1), which corresponds to the most favored D-π-A configuration of DSSC sensitizers [21]. Such an alignment empowers the applicability in DSSCs as it favors the intramolecular charge transfer (ICT) during the molecule’s excitation, which might be converted to photocurrent if the sensitizer is properly oriented on the surface of the semiconductor [22]. Surprisingly, there are no reports describing the use of 2-thiohydantoin moiety as an anchor for DSSC sensitizers. Therefore, we decided to investigate the possibility of using its derivatives as sensitizers. Furthermore, the studies of the compounds presented in Figure 1 allowed us to explain the role of aromatic π-spacer and the carboxymethyl substituent in position N-3 of the heterocycle in the light conversion process.

## 2. Results and Discussion

### 2.1. Theoretical Considerations

**Figure 2:**
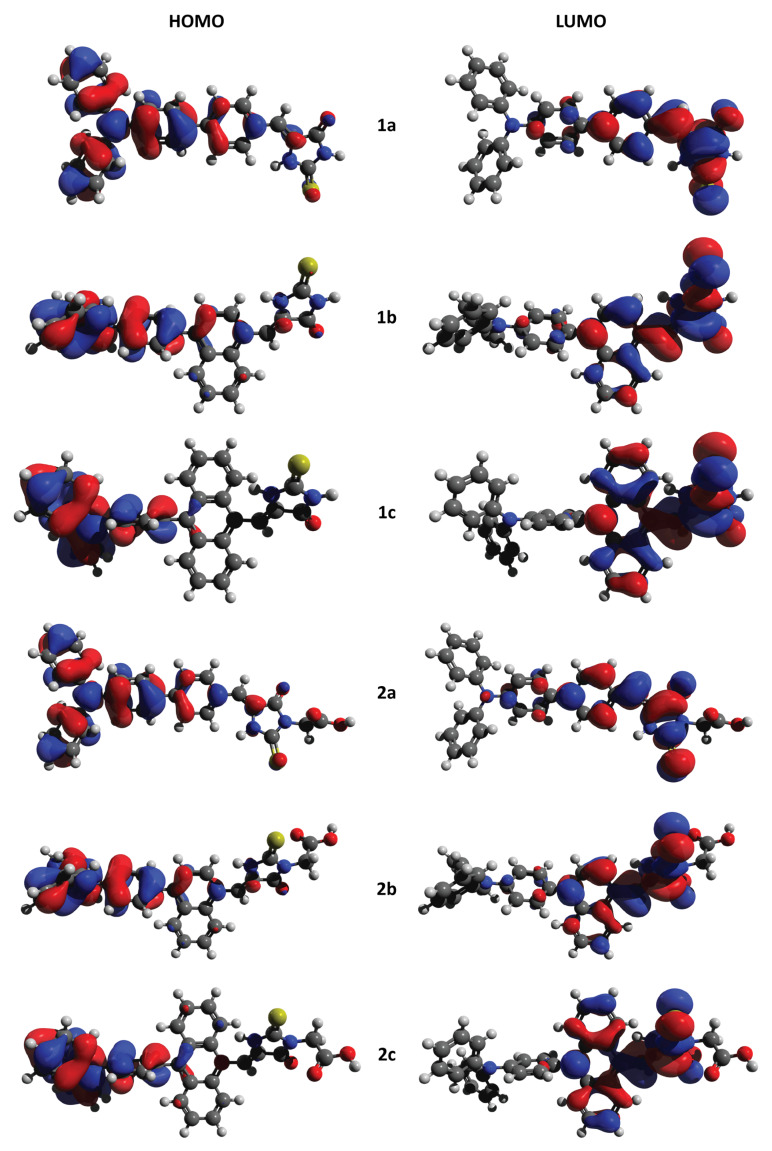
Visualization of HOMO and LUMO orbitals calculated for the examined molecules.

Nowadays, computational methods constitute a powerful tool enabling prediction of various properties of compounds, even prior to their time- and work-consuming synthesis. In particular, the DFT method has proved to be very useful in the modeling of electronic structures of many DSSC sensitizers, providing an initial guess about molecular geometries, distribution and energy levels of frontier orbitals, dipole moments, and absorption spectra [23,24,25]. Therefore, we started our studies with the prediction of the examined compounds’ electronic structures. Initially, the ground state structures of **1a**–**2c** were optimized using a B3LYP functional and 6-31+G** basis set with the polarizable continuum model (PCM) to emulate the dichloromethane environment [26,27]. Atom coordinates are included in Supplementary Material (SM, Tables S1–S6), whereas the most important parameters for discussion are presented in Table 1. It was predicted that the π-aromatic spacer should significantly affect the relative conformation of the dyes’ aromatic moieties. The compounds bearing 1,4-phenylene linker (**1a**, **2a**) were the most planar ones, with a maximum dihedral angle between the spacer and the heterocycle plane of 18.0°, and 32.8° between the spacer and the adjacent 1,4-phenylene group of the triphenylamine moiety. With the increasing size of π-aromatic spacer, the predicted dihedral angles reached up to 53.4° and 83.1°, respectively. In contrast, the molecule’s dipole moment decreased with increasing spacer size. The dihedral angles were only slightly affected by the substituent in position N-3 of the heterocycle, while the presence of a carboxymethyl group caused a reduction in the dipole moment (compare **1a**–**c** with **2a**–**c**). Therefore, it should be expected that the changes in molecular geometry should have an impact on the dyes’ electronic properties and their performance in the devices. Indeed, the calculated frontier molecular orbitals distribution were found to be affected by the molecular geometry (see Figure 2). In the investigated compounds, HOMOs were located mostly on the triphenylamine moiety, while LUMOs were mainly present on the π-aromatic spacer and the heterocycle. Such a distribution is beneficial for effective dye operation since, during the photoexcitation, the electrons are shifted from triphenylamine towards the heterocyclic moiety, which is expected to act as an acceptor group located close to the anode surface. As a result, the electrons should be collected by the TiO_2_ anode, and the holes should recombine with the electrons donated from the cathode with the aid of the electrolyte, thus resulting in a photocurrent. However, as the dihedral angles increased, the distribution of HOMO over the π-aromatic spacer and LUMO distribution over triphenylamine moiety decreased. Consequently, the HOMO–LUMO overlap also decreased, which might hamper the electron transfer during photoexcitation. Similarly, electron transfer can be suppressed in **2a**–**c** as the methylene group was found to prevent the conjugation of the heterocycle and carboxyl group, the latter being a well-known group anchoring to the TiO_2_ surface [28].

The calculated frontier molecular orbital energy levels are presented in Table 1. Unexpectedly, their values were negligibly affected by the variation of the π-aromatic spacer size, giving the HOMO and LUMO energy values within −(5.54–5.59) eV and −(2.80–2.83) eV, respectively. Therefore, their bandgap is expected to be very similar, resulting in similar light absorption properties. The most important fact is that the calculated HOMO energy levels are more negative compared to the reduction potential energy of the I_3_^−^/I^−^ electrolyte (−4.80 eV), and the LUMO energy levels are more positive in reference to the conduction band of TiO_2_ (−4.0 eV), both facilitating the expected transport of the photogenerated charge carriers [29].

Finally, the TD-DFT method was applied to predict the absorption spectra of the examined compounds and identify the most important electronic transitions. On the basis of the frontier molecular orbitals distribution, it was evident that the molecules should show high transition dipole moments. Therefore, the CAM-B3LYP method comprising long-range correction was applied at this stage of quantum calculations [30]. As a result, two major bands were predicted for each compound (see Table 1). The strong band characterized by λ_max_ within a 398–438 nm range was identified as an intramolecular charge transfer (ICT), a transition from triphenylamine moiety to π-aromatic spacer and the heterocycle. The second shorter-wavelength band was attributed to π–π* transitions in the aromatic moieties within the molecules. Both bands undergo bathochromic shifts with the change of the spacer from 1,4-phenylene to 1,9-anthracenylene, which is probably caused by the expansion of the conjugation.

### 2.2. Electrochemistry

**Figure 3:**
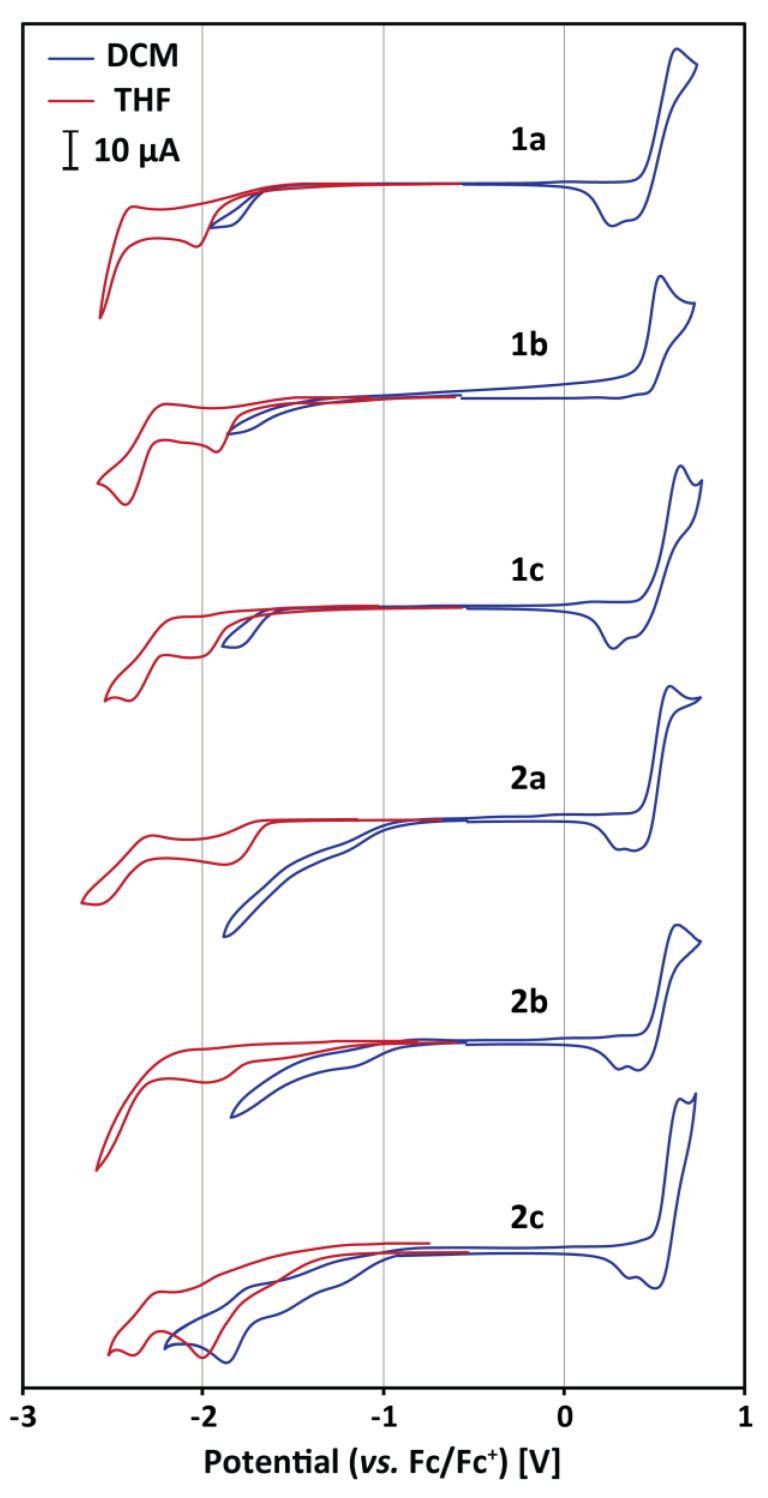
Cyclic voltammetry of 2 × 10^−3^ M solutions of the studied compounds in DCM/(n-Bu)_4_NPF_6_ (blue curves) and THF/(n-Bu)_4_NPF_6_ (red curves); scan rate 0.1 V/s.

After obtaining promising theoretical predictions, we moved to the experimental characterization of the examined compounds. In the beginning, the cyclic voltammetry (CV) experiments were carried out in DCM/Bu_4_NPF_6_ solution to determine the actual ionization potentials (IP) that correspond to the HOMO (E_HOMO_ = −IP) energy level, and electron affinities (EA) that correspond to the LUMO (E_LUMO_ = −EA) energy level. The obtained voltammetry curves are shown in Figure 3, while the numerical data are summarized in Table 2. The measured onsets of oxidation potential (E_ox_) using ferrocene as an internal standard (0.46 V vs. SCE) were very similar and ranged from 0.44 to 0.49 V. These values correspond to IP in the range from 5.54 to 5.59 eV and are related to the HOMO located mainly on the triphenylamine moiety. The determined IPs were only slightly varied, probably due to weak conjugation of the triphenylamine group with the other parts of the molecules, as shown by the theoretical calculations. Very close values have been reported for similar triphenylamine-based dyes [31,32,33,34,35]. It is noteworthy that the experimental values correlate very well with the theoretically calculated ones. The predicted HOMO energy levels were also negligibly varied (maximum spread 0.04 eV), but, in general, by ~0.2 V more positive than the determined values. In addition, the shape of the voltammograms and the deposition of an electroactive product on the working electrode surface indicated the occurrence of the dimerization/polymerization process. This phenomenon has been reported for the other phenylamine derivatives [36].

The reduction process of the studied compounds by CV in THF/Bu_4_NPF_6_ and DCM/Bu_4_NPF_6_ solutions was investigated. THF was additionally chosen because of a wider potential window in the cathodic regime compared to DCM. The measurement revealed a complex irreversible reduction process with significant differences in both solvents. It is shown in Figure 3 that the onsets of the reduction potentials are more negative in THF than in DCM. A comparison of the cathodic and anodic current recorded in the same scan in the DCM environment has proven that these processes are not charge-equivalent. Getting into the details, the cathodic current was at least two times lower than the anodic current. While the oxidation of triphenylamine moiety is a one-electron process, more than one dye molecule must be involved in the one-electron reduction process. The explanation of this phenomenon, as well as differences in behavior in different solvents, is the formation of thiohydantoin aggregates. The formation of such associates has already been predicted theoretically and observed for the molecules of similar structures [37,38,39]. The formation of these aggregates must be dependent on the polarity of the medium in which they are dissolved, which might be the reason for the difference between the voltammograms measured in DCM and THF. Since the measurements revealed that the reduction process is very complex and the reduction onsets are much different, while the theoretical calculations suggested that the LUMO energy levels should be very similar, we decided not to estimate the EA.

### 2.3. Optical Characterization

**Figure 4:**
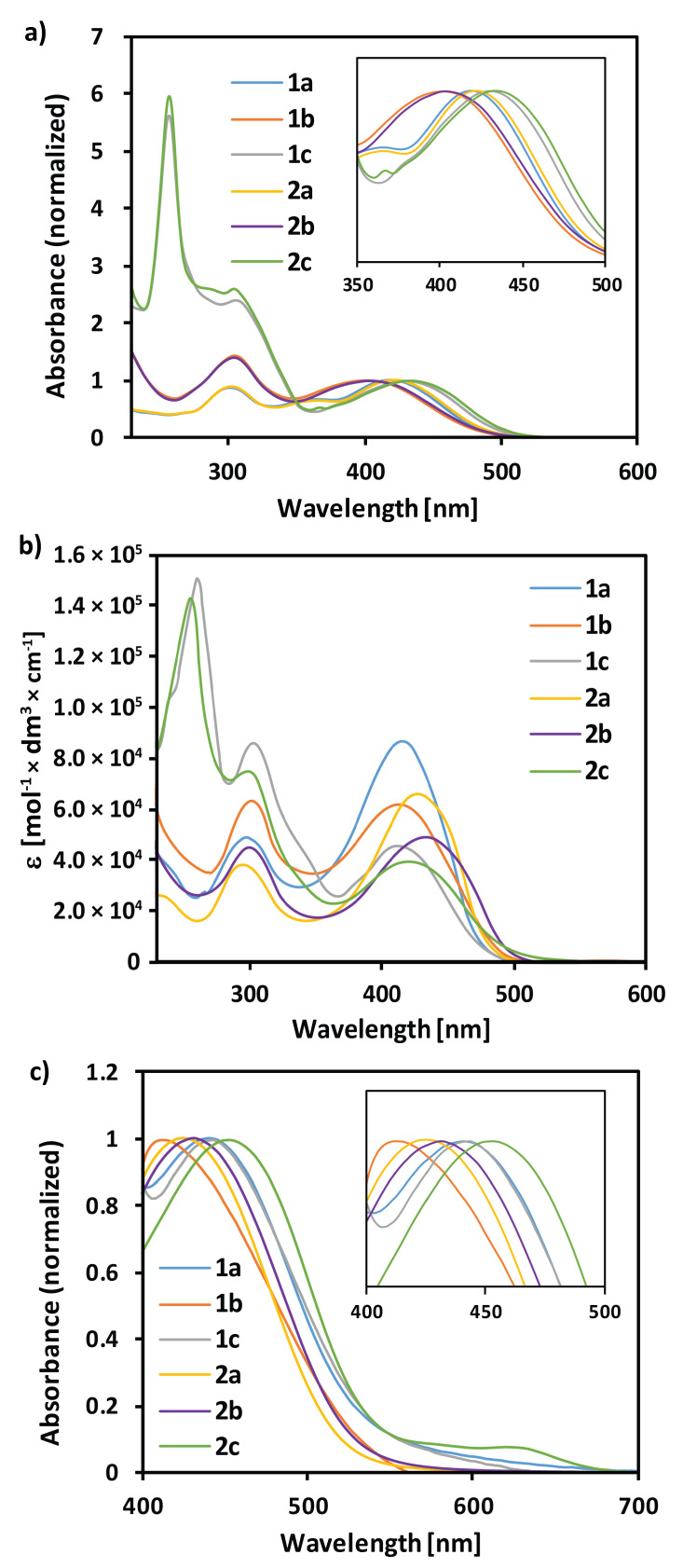
Absorption spectra of **1a**–**2c** (**a**) in DCM (normalized, inset: zoom on the visible absorption range); (**b**) in EtOH; (**c**) adsorbed on TiO_2_ (normalized, inset: zoom on the visible absorption range).

Absorption spectroscopy is a very useful method for the characterization of active species for optoelectronic devices. In order to verify the validity of the theoretical model, the absorption spectra of the examined compounds were initially measured in DCM. The normalized spectra are presented in Figure 4a, and the extracted wavelengths are compiled in Table 3. All the spectra were characterized by a strong ICT band in the visible range, with maxima within 402–434 nm. These values were very accurately predicted, except for compounds **1a** and **2a**. Unexpectedly, the compounds bearing 1,4-phenylene π-spacer showed ca. 20 nm bathochromic shift compared to the derivatives bearing 1,4-naphthalenylene moiety. The second band, typical of all the compounds, was observed slightly above 300 nm. These bands were also predicted by the DFT method and assigned to π–π* transition of the aromatic moieties, with the difference between the theoretical and experimental data up to 26 nm. The very close maxima of these bands suggest that the latter must originate from a structural motif common to all the compounds, probably the triphenylamine moiety. Finally, a very strong absorption was recorded for compounds **1c** and **2c** at 256 nm, which is the effect of π–π* transitions characteristic of 1,9-antrhracenylene moiety. It is worth emphasizing that the compounds with the same π-spacer in the structure (**1a** and **2a**, **1b** and **2b**, **1c** and **2c**) exhibited almost identical maximum absorption wavelength, so the incorporation of the carboxymethyl group in **2a–c** did not result in the meaningful difference. The optical bandgaps were calculated on the basis of the absorption onsets in DCM. All the bandgaps were only slightly varied and ranged from 2.46 to 2.54 eV.

The LUMO energy levels (E_LUMO opt_) were calculated by the addition of the optical bandgaps (E_g opt_) to the HOMO energy levels measured by CV in DCM (E_HOMO_ = −IP). The obtained values did not vary significantly and oscillated around −3 eV. The experimentally determined LUMO energy levels correlated well with the predicted ones but were generally overestimated by about 0.2 eV. However, if we consider that the predicted HOMO energy levels oscillated around −5.3 eV, while LUMO around −2.8 eV, both overestimated by about 0.2 eV, we obtained predicted bandgaps oscillating around 2.5 eV, very close to the measured E_g opt_. Nonetheless, no correlation was observed between the molecule configuration twist and λ_ICT,_ which requires explanation. In general, the twist of configuration ought to cause a reduction of conjugation and an increase in the bandgap (lowering of λ_ICT_). However, **1c** and **2c,** characterized by the most twisted configuration, showed the longest λ_ICT_ in their series. It is important to keep in mind that not only the twist itself should be considered, but also the electronic effect of the aromatic spacer extension. As we can see in Figure 2, the LUMOs are distinctly located on the heterocycle and the aromatic spacer, regardless of the dihedral angle between these two moieties. This should imply stabilization of the LUMO energy level if the aromatic spacer is extended, as a consequence of increased conjugation. This trend was observed both for the measured E_LUMO opt_ and calculated E_LUMO teo_, but it was slightly underestimated by the B3LYP method. We believe that the stabilization of LUMO and the related reduction of the bandgap are the reasons why **1c** and **2c** equipped with 1,9-anthracenylene spacer showed the longest λ_ICT_. On the other hand, the less extended aromatic spacer should stabilize the LUMO energy level to a lesser degree, but simultaneously it should make the molecule to be less twisted, as in the case of **1a–b**, and **2a–b**. The more planar the structure, the more intensive the interaction between HOMO and LUMO levels, implying lowered bandgap (increased λ_ICT_) in the case of **1a** and **2a**. Therefore, we believe that **1b** and **2b** showed the shortest λ_ICT_ due to the sufficient configuration twist to prevent HOMO–LUMO interaction, and the presence of 1,4-naphthalenylene spacer having a less stabilizing effect on the LUMO than 1,9-anthracenylene moiety. Hopefully, the LUMO energy levels turned out to be more positive compared to the conduction band of TiO_2_ (−4.0 eV), so the electron transfer from the photoexcited dyes to the anode should be favored.

Absorption spectra are known to be affected by the solvents used to solubilize the analyzed compounds, which is known as the solvatochromic effect. The latter is particularly pronounced for the compounds with ICT character of electronic transitions. However, the magnitude and direction of the shift depend essentially on the electrical permittivity of the environment, so the absorption of the dye adsorbed on the TiO_2_ surface might be different from that in a nonpolar solvent. Therefore, we chose ethanol (EtOH) as a better solvent than DCM for molar attenuation coefficient determination, since it would better reproduce the real conditions inside the DSSCs. In addition, the anodes’ sensitizations were conducted in EtOH solutions. As shown in Figure 4b, the examined compounds varied in their absorption properties. The most intensive absorption in the visible region was observed for **1a** and **2a**, while the weakest for **1c** and **2c**. This difference is probably caused by the predicted conformation twist in the compounds equipped with 1,9-anthracenylene spacer that hampers ICT in contrast to the most planar molecules bearing a 1,4-phenylene linker. It is noteworthy that the molar absorption coefficients were higher than those of similar organic dyes and much higher than those of the Ru-based dyes [32,40]. Unexpectedly, the molar absorption coefficients of the ICT band were always lowered when the carboxymethyl group was introduced in position N-3 of 2-thiohydantoin. This decrease can be attributed to the lower transition dipole moments in the compounds of series **2** compared to those of series **1**, which is reflected in the reduction of the ground state dipole moment. In the absorption measurements in DCM, the spectra of **1c** and **2c** in ethanol were dominated by the absorption of the anthracenylene moiety. The numerical parameters obtained from the absorption measurements are presented in Table 3. As shown, the maximum wavelengths of the second band changed only by a few nanometers, which confirms their character typical of π–π* transitions. On the contrary, the ICT maxima were shifted by up to 29 nanometers, which might be related to the polar and protic nature of EtOH. Interestingly, both the bathochromic and hypsochromic shifts were observed. Such an observation indicates that the molecules adapt different geometries in DCM and EtOH. This effect must be caused by the variation of the linker because the compounds equipped with 1,9-anthracenylene exhibited a hypsochromic shift, compounds with 1,4-naphthalenylene showed a bathochromic shift, and derivatives with 1,4-phenylene unit exhibited both negligible bathochromic and hypsochromic shift.

Subsequently, we examined the effect of the dyes’ adsorption on the TiO_2_ on the absorption properties of the system. TiO_2_ nanoparticles in the form of 10 µm layers were deposited on the FTO substrates, then the layers were exposed to the dyes’ solutions under the standardized conditions, and the diffuse reflectance spectroscopy measurements were performed. To reproduce the DSSC conditions as well as possible, the active layers were prepared in exactly the same manner as the anodes during the solar cells’ fabrication process. Therefore, ethanol was used as the medium from which adsorption was carried out since it was proved that, from among other solvents, its use ensures the highest efficiency of the photovoltaic cells [41,42,43]. In addition, chenodeoxycholic acid (CDCA) was introduced into the impregnation system to prevent aggregation of the dyes’ molecules, which could lead to the improvement of the devices’ efficiency [44]. The normalized spectra are presented in Figure 4c, while the ICT absorption maxima are given in Table 3. As evidenced, all the examined compounds adsorbed on the TiO_2_ surface thanks to the interaction of the polar heterocyclic anchor itself or supported by carboxymethyl group, with the anode surface [23]. The visible-region bands of all the dyes showed a bathochromic shift after being adsorbed on TiO_2_, compared to the measurements in DCM solution, and almost the same was observed with respect to the EtOH solutions. The shifts might be attributed to different conformations of the dyes’ molecules, their deprotonation, or the dye aggregation, all caused by the interaction with the TiO_2_ surface [45,46]. However, CDCA was incorporated as the co-adsorbent due to its known ability to prevent dyes’ aggregation, so the last explanation is unlikely. The aggregation was only observed in **2c**, as evidenced by the appearance of a band at around 630 nm.

### 2.4. Photovoltaic Properties

**Figure 5:**
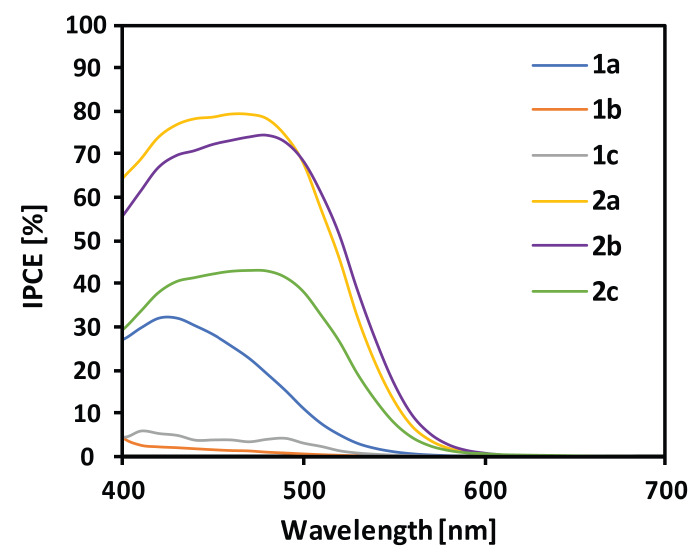
Internal power conversion efficiency chart.

The performance of the fabricated cells was tested under standard AM 1.5G solar irradiation, and the obtained incident photon to current conversion efficiency (IPCE) spectra are presented in Figure 5. In general, the IPCE spectra were broadened relative to the absorption spectra of the dyes deposited on the TiO_2_ surface. This implies that the devices can convert a broader range of solar radiation into electric energy. The best conversion showed the devices based on **2a** and **2b**, with IPCE reaching even up to 80%. On the contrary, **1b** and **1c** based cells were found to be the least efficient, probably due to the poor photovoltaic action of the dyes, as their IPCEs do not resemble the UV-Vis spectra. Interestingly, the compounds bearing 1,4-phenylene spacer exhibited the highest IPCE within the series **1a–c** and **2a–c**, which must be caused by the most planar geometries of **1a** and **2a**, as it is favorable for enhanced ICT efficiency. It is noteworthy that the device based on **2b** showed almost the same IPCE as **2a**, while no similar trend was observed for the **1b** and **1a** couple. Compounds **1a–c** and **2c** showed much narrower IPCE action spectra in reference to their absorption spectra on TiO_2_. In the case of **2c**, the reason is that the broad long-wavelength band related to the aggregation is not active in the photoconversion process, whereas, in the case of **1a**–**c**, the poor performance of the cells limited the detection of IPCE above ~530 nm making the impression that the IPCE action spectra are narrower due to the scaling of the chart.

**Figure 6:**
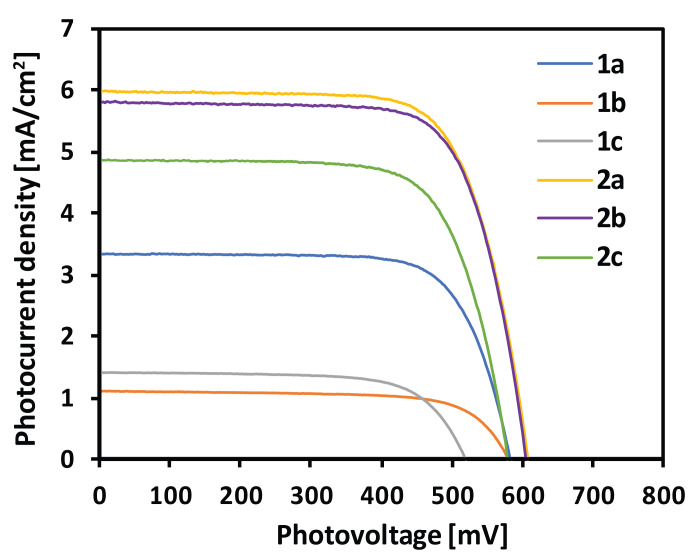
J–V characteristics of the fabricated solar cells.

Figure 6 presents the photocurrent density–voltage curves of the DSSCs based on the examined compounds. The plots confirmed the results of the IPCE measurements, proving the best performance of **2a** and slightly less of **2b**. The parameters of the fabricated devices containing the examined dyes are listed in Table 4. The highest achieved short-circuit photocurrent (J_SC_) was close to 6 mA/cm^2^, and the open-circuit voltage (V_OC_) was close to 600 mV. The worst parameters showed the devices based on **1b** and **1c**, which generated only up to 1.4 mA/cm^2^ and were characterized by V_OC_ up to 592 mV. The power conversion efficiencies (PCE) for the latter devices were similar and close to 0.5%, and also the lowest from among the examined dyes. The highest performance in the series **1a–c** showed dye **1a**, giving almost threefold improvement with regard to the others. Compounds **2a–c** showed significantly better photovoltaic performance compared to their counterparts not equipped with a carboxymethyl group. The highest PCE of 2.59% was recorded for dye **2a**, and decreased with the extension of the π-aromatic linker size to the value of 2.00% in **2c**.

All the DSSCs were characterized by very close values of V_OC_ (except for **2c**) and the fill factor (FF) in the range 69.2–72.5%, so the differences in the PCEs must be attributed to the variation of J_SC_. According to literature, the photocurrent is strongly affected by the dye loading [47]. Since the carboxyl group is commonly employed as an anchor improving dyes’ adsorption properties, we assumed that the presence of the carboxymethyl group that differentiates series **1a**–**c** and **2a**–**c** must have a crucial impact on the dyes’ adsorption properties and thus modulates the cells’ efficiencies [1,2]. Therefore, the dyes’ loadings were determined by desorption in basic conditions followed by absorbance measurement and related to PCE. The obtained values are listed in Table 4. Indeed, the presence of an additional substituent in position N-3 significantly boosted the dyes’ concentration on the TiO_2_ surface, even up to 80 times for compounds **1a** vs. **2a**. In series **2a**–**c**, the dye loading decreased as the size of the linker increased, which must be attributed to the twisted conformation and the space occupied by the molecule, thus causing more loose coverage of the surface. The variation of dye loading might have its own contribution to the PCE trend in series **2a**–**c**. On the other hand, the same trend in dye loading was not observed for series **1a**–**c** since **1b** breaks out of the rule. Compounds of this group showed a significantly lower affinity to adsorption on the TiO_2_ surface. Among them, **1b** was characterized by the highest dye loading, which suggests its more dense packing as the adopted conformation was different than that predicted in the solution, or the dye aggregation. This might be the cause of the outstanding parameters of **1b**, such as the longest electron lifetime and the lowest J_SC_, PCE, and λ_ICT_ on TiO_2_ (see Table 3 and Table 4). However, **1a** exhibited unusually high performance with respect to its surface loading. Even dyes of much more complex structure and characterized by similar surface loading did not show such a high efficiency [48,49]. Moreover, the dyes of similar structure equipped with hydantoin, 2,4-thiazolidinedione, and rhodanine-3-acetic acid acceptor groups showed inferior performance relative to **1a** [32,50].

### 2.5. Electrochemical Impedance Spectroscopy

**Figure 7:**
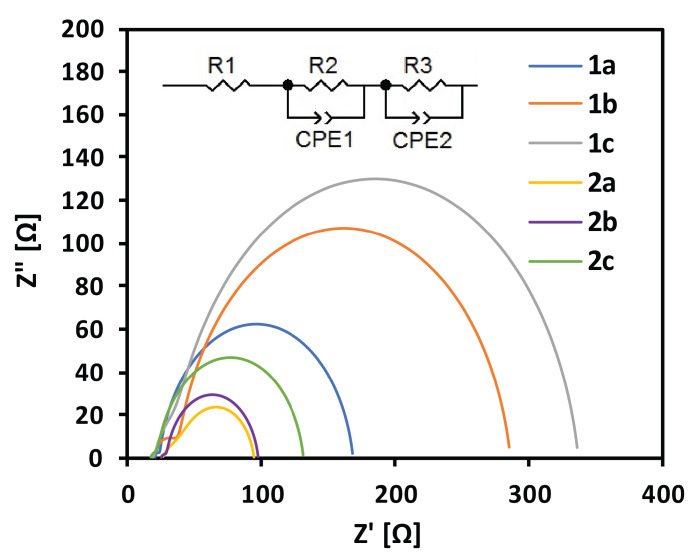
Fitted Nyquist plots of impedance spectra of the DSSC devices.

Electrochemical impedance spectroscopy is a useful tool to elucidate the electron injection and recombination processes in DSSCs [51,52]. Figure 7 presents the fitted Nyquist plots of impedance spectra of the cells sensitized with the studied dyes and an electrical equivalent circuit used for data fitting. The values of the obtained resistance parameters and estimated excited electron lifetimes are summarized in Table 4. The ohmic serial resistance (R_1_) observed at a high frequencies region represents the FTO and external circuit resistance. It was found to be similar for all investigated cells and its values are typical of DSSC measurements [53,54]. At high frequencies, the resistance of the redox charge transfer process on the Pt electrode of the solar cell values was observed (R_2_), and it showed a more significant variation in our case than R_1_ values. This may be related to the discontinuities in the Pt film and/or the differences in the film thickness, which is typical of imperfect “hand-made” counter electrodes [55,56]. As the R_1_ and R_2_ values are less important according to the presented results, the electron transfer resistance at the TiO_2_/dye/electrolyte interface (R_3_) strongly depends on the sensitizing dye structure, especially on the aromatic moiety structure located between the D and A part of the dye molecules, and corresponds to the overall cell performance. In general, the higher the R_3_ value, the lower the efficiency of the investigated cell. The observed relation between R_3_ and PCE showed that the bulkier structure of the π-aromatic linker prevented an efficient electron injection process, resulting in the observed decrease in cell efficiencies.

**Figure 8:**
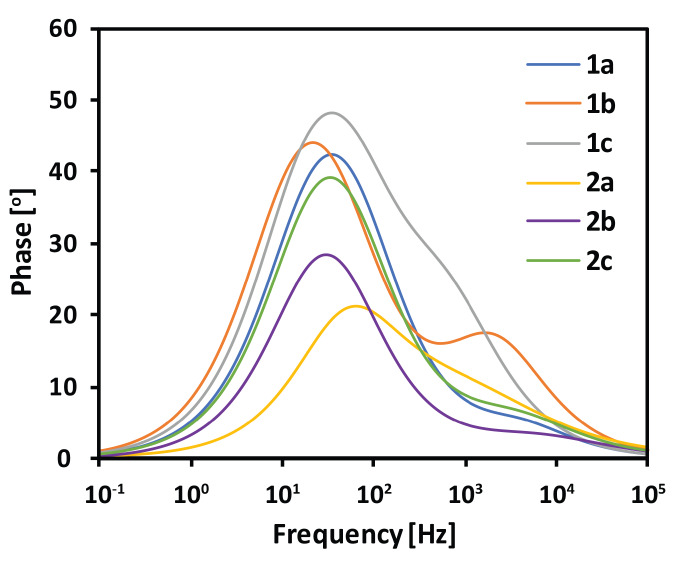
Bode phase plot under V_OC_ forward bias conditions.

The EIS spectra were measured under the standard simulated solar light, at V_OC_ preventing the current flow through the external circuit. Under these conditions, the photogenerated electrons injected into the semiconductor conduction band have to recombine with the electrolyte on the TiO_2_/dye/electrolyte interface [57,58]. Consequently, the estimated electron lifetime values (τ) may be calculated according to the equation τ = (2π*f*)^−1^, using the frequency value at the maximum phase angle (*f*) from the low-frequency region of the Bode phase plot presented in Figure 8. Longer electron lifetime at the TiO_2_/dye/electrolyte interface is desired since it implies a more efficient prevention of charge recombination and a more plausible generation of the photocurrent [33,59]. From among the examined dyes, compounds **1b** and **2b** exhibited the longest τ (see Table 4), while **2a** the shortest. In conclusion, the compounds equipped with 1,4-naphthalenylene linker prevented electron recombination most efficiently. Surprisingly, the cell comprising **2a** was characterized by the highest PCE and exhibited the shortest τ, while the cell based on **1b** showed the lowest PCE and the longest τ. Furthermore, no correlation between V_OC_ and τ was observed, contrary to literature reports [2,33,60]. Interestingly, **2a** showed the highest V_OC_ among the tested dyes and the shortest electron lifetime, which is rather unusual. In general, the rise in electron lifetime should imply electron concentration in the semiconductor to be higher, causing an increase in the quasi-Fermi energy level of TiO_2_. This ought to result in a higher V_OC_, but it is not always observed [61], as in the case of **2a**. Therefore, we came to the conclusion that the electron lifetime was not a determinant for the device performance and the other factors were more important. On the basis of the above studies, we suggest that the best DSSC performance of **2a** is mainly attributed to its highest dye loading and the most planar structure facilitating ICT excitation, both factors responsible for the increase in electron density in the semiconductor, while the short τ is not critical for its performance.

## 3. Materials and Methods

### 3.1. Materials

All the dyes were synthesized according to the previously published procedures [20]. The dyes purity was determined to be at least 97% by HPLC. All the other chemicals were of sufficient grade and used without further purification. Acetic acid, acetone, and anhydrous EtOH were obtained from Avantor Performance Materials (Gliwice, Poland), anhydrous acetonitrile and HPLC grade DCM from Sigma Aldrich (St. Louis, MO, USA), and anhydrous THF from Acros Organics (Fair Lawn, NJ, USA). 1-methyl-3-propylimidazolium iodide, lithium iodide, iodine, 4-*tert*-butylpyridine, titanium(IV) chloride, hexachloroplatinic acid, α-terpineol, ethylcellulose, and CDCA were received from Sigma Aldrich, while tetrabutylammonium hexafluorophosphate was from TCI Chemicals (Tokyo, Japan). TCO22-7 FTO glass substrates were obtained from Solaronix, P25 Aeroxide titania nanopowder from Evonik (Essen, Germany).

### 3.2. Theoretical Calculations

All the calculations were performed with the use of Gaussian 09 software (Version D.01; Gaussian, Inc.: Wallingford, CT, USA) [62]. The molecular geometries in the ground state were optimized at the B3LYP/6-31+G** level using PCM to simulate the DCM environment [26,27]. Frequency calculations were performed to confirm the optimization of the stationary point. The dihedral angles and dipole moments were obtained using the ground state geometries. The frontier molecular energy levels were calculated using the same level of theory with the optimized structures. Finally, the time-dependent DFT (TD-DFT) calculations were performed at the CAM-B3LYP/6-31G** level in DCM to get the excitation energy for the 50 lowest singlet–singlet transitions. The obtained excitation energies and the oscillator strengths were used to simulate the absorption spectra with the aid of GaussView 5.0 software (Version 6; Semichem Inc.: Shawnee Mission, KS, USA) [63].

### 3.3. Electrochemical Studies

CV measurements were performed on an Autolab PGSTAT M101 (Metrohm, Herisau, Switzerland). Measurements were carried out on a classic three-electrode assembly. Pt wire was used as a working electrode, Pt spiral as a counter electrode, and silver wire as a pseudo-reference electrode. The scan rate was 100 mV/s. Potentials were calibrated by ferrocene as an internal standard. Solutions of 0.2 M tetrabutylammonium hexafluorophosphate in anhydrous DCM or THF were used as a supporting electrolyte. The concentration 2 × 10^−3^ M of the studied compounds was used. The solutions were deoxidized by argon bubbling prior to the measurement. IP was estimated from equation IP = |e^−^|(5.1 + E_ox_).

### 3.4. UV-Vis Spectroscopy

Absorption measurements in DCM and EtOH solutions were carried out on a Cary 50 (Varian, Palo Alto, CA, USA) and Cary 60 (Agilent Technologies, Santa Clara, CA, USA) spectrometers. Diffuse reflectance UV-Vis spectra were recorded on Cary 5000 spectrometer (Varian) equipped with a 100 mm integrating sphere. The samples for the diffuse reflectance measurements were prepared by the immersion of TiO_2_-coated FTO glass plates in a solution of the dye (3 × 10^−4^ M) and CDCA (1 × 10^−3^ M) in anhydrous ethanol overnight, subsequent washing with pure solvent, and dried in air. The dye loadings were determined by the immersion of the TiO_2_-coated plates with adsorbed dyes in 5 mL of 1 M ammonia solution in EtOH, followed by the UV-Vis analysis of the solution containing the desorbed dye. The measured absorbances were compared with the calibration curves obtained for various concentrations of the dyes. Using the latter, the molar absorption coefficients were also determined. Five plates were analyzed for each dye, and the results were averaged. The spectral data processing was performed with Spectragryph software [64].

### 3.5. Solar Cells Fabrication

A TiO_2_ paste was prepared using an already described method [65], according to the following procedure: 3 g of TiO_2_ nanopowder was mixed with 0.5 mL of acetic acid and 20 mL of ethanol and kept in an ultrasonic bath for 3 h. A solution containing 1.5 g of ethyl cellulose and 10mL of α-terpineol in 13.5 g of ethanol was prepared and then added to the former suspension. The mixture was sonicated for 1 h and then magnetically stirred overnight. Ethanol was slowly removed in a rotary evaporator, and the obtained paste was ready-to-use. TiO_2_ films were deposited by the “*doctor blade*” technique on the FTO substrate using a Scotch tape mask of 62.5 µm thickness. Finally, the electrodes were calcined in an oven at 723 K for 2 h. After cooling down, working electrodes were immersed in 40 mM TiCl_4_ aqueous solution and kept at 343 K for 1 h followed by washing with deionized water and ethanol, dried in a hot air stream, and calcined in the oven at 723 K for 30 min. After cooling down, the electrodes were immersed in 3 × 10^−4^ M ethanolic solution of the studied dyes containing 1 × 10^−3^ M of CDCA and kept in the dark at room temperature for 24 h. The time of the dyeing was recognized to be sufficient for the equilibrium of adsorption to be settled [66,67]. After dye adsorption, the electrodes were washed with absolute ethanol and dried in a hot air stream. Platinum-coated FTO was used as a counter electrode and prepared as follows: an ethanolic solution of H_2_PtCl_6_ containing 23 g/L Pt was spread on the FTO substrate using cellulose tissue, dried and calcined in the oven at 723K for 30 min. The typical cell was assembled using a 25 μm thick, hotmelted ionomeric foil Meltonix (Solaronix, Aubonne, Switzerland) as a sealant and a spacer between the electrodes. The electrolyte contained 0.6 M 1-methyl-3-propylimidazolium iodide, 0.1 M lithium iodide, 0.05 M iodine, 0.5 M 4-*tert*-butylpyridine in acetonitrile and its composition was based on the literature report with little modification [39]. The electrolyte was injected within two holes predrilled in the counter electrode. The final sealing was realized with the use of a hot melted sealant and a microscope cover slide. The typical active area of the obtained DSSC was approximately 0.125 cm^2^.

### 3.6. Photovoltaic Characteristics

The photovoltaic properties of the cells were measured under irradiation from Sun 2000 class A solar simulator (Abet Technologies, Milford, CT, USA) equipped with an AM 1.5G filter, with the light intensity adjusted to 100 mW/cm^−2^ using a silicon reference cell equipped with KG5 filter (ReRa Systems, Nijmegen, The Netherlands). J–V curves were recorded on an Autolab PGSTAT 302N (Metrohm). IPCE spectra for the studied solar cells were recorded using an Autolab M101 PGSTAT Galvanostat (Metrohm) coupled to a photoelectric spectrometer equipped with a solar simulator (Photon Institute, Kraków, Poland).

### 3.7. Electrochemical Impedance Spectroscopy

Electrochemical impedance spectra were recorded under standard AM 1.5G simulated solar irradiation, obtained from Sun 2000 class A solar simulator (ABET Technologies, Milford, CT, USA). The frequency was measured on Autolab PGSTAT 302N (Metrohm) equipped with the FRA32 module, in the range from 0.1 Hz to 100 kHz, under V_OC_ forward bias conditions, and with V_AC_ = 10 mV.

## 4. Conclusions

In summary, a comprehensive characterization of a series of dyes composed of triphenylamine and 2-thiohydantoin or 3-carboxymethyl-2-thiohydantoin moieties linked with 1,4-phenylene, 1,4-naphthalenylene and 1,9-anthracenylene spacers, oriented towards their application in DSSC, was performed. The theoretical calculations, electrochemical measurements, absorption spectroscopy, and electron impedance spectroscopy analysis, and, finally, characterization of the assembled cells allowed a thorough study proving that 2-thiohydantoin might act as an acceptor and an anchoring group. Surprisingly, the most efficient dye was found to be **2a**, the compound characterized by the strongest bactericidal properties from among the compounds examined in our earlier studies. The best device showed V_OC_ = 607 mV, J_SC_ = 5.97 mA/cm^2^, FF = 71.4%, and PCE = 2.59%, while the cell containing **2b** exhibited very close parameters. We concluded that the cells’ performances were determined by the dye loading and ICT molecular absorption coefficients, both affected by the differences in the chemical structure of the dyes. The presence of the carboxymethyl group in position N-3 of the heterocycle significantly improved the dye loading, which was further modulated by the aromatic linker size. Additionally, we found that, with increasing π-spacer size, the ICT efficiency decreased, resulting in the higher performance of the dyes bearing 1,4-phenylene spacer as the molecules of most planar geometries. We believe that our findings for relatively small molecules for DSSC might be useful for further development of renewable energy devices.

## Figures and Tables

**Table 1:** Predicted molecular and electronic properties of the studied compounds.

Compound	π-Spacer/ 1,4-Phenylene Dihedral Angle [^o^]	π-spacer/ Heterocycle Dihedral Angle [^o^]	Dipole Moment [D]	E_HOMO teo_ [eV]	E_LUMO teo_ [eV]	λ_ICT teo_ [nm]	λ_π–π* teo_ [nm]
**1a**	32.8	16.0	8.2	−5.34	−2.80	398	275
**1b**	56.2	34.8	7.4	−5.35	−2.80	400	285
**1c**	83.1	53.4	6.8	−5.38	−2.81	431	290
**2a**	32.7	18.0	6.6	−5.34	−2.81	399	276
**2b**	56.2	34.2	5.8	−5.35	−2.82	401	285
**2c**	76.4	53.0	5.2	−5.37	−2.83	438	293

**Table 2:** The numerical data of the cyclic voltammetry measurements.

Compound	E_ox DCM_ [V]	IP [eV]	E_HOMO_ [eV]	E_red DCM_ [eV]	E_red THF_ [eV]
**1a**	32.8	16.0	8.2	−5.34	−1.86
**1b**	56.2	34.8	7.4	−5.35	−1.79
**1c**	83.1	53.4	6.8	−5.38	−1.84
**2a**	32.7	18.0	6.6	−5.34	−1.65
**2b**	56.2	34.2	5.8	−5.35	−1.14
**2c**	76.4	53.0	5.2	−5.37	−1.34

**Table 3:** Optical properties of the studied compounds.

Compound	λ_ICT_ [nm]	λ_π–π*_ [nm]	λ_abs onset_ [nm]	E_g opt_ [eV]	E_LUMO opt_ [eV]	λ_ICT_ [nm]	λ_π–π*_ [nm]	ε_ICT_ [mol^−1^ dm^3^ cm^−1^]	λ_ICT_ [nm]
	DCM					EtOH			TiO_2_
**1a**	419	301	488	2.54	−3.01	417	298	87100	441
**1b**	402	305	486	2.55	−2.99	413	301	61600	416
**1c**	431	306	501	2.47	−3.12	413	303	45700	443
**2a**	422	302	492	2.52	−3.02	426	296	66100	425
**2b**	404	304	488	2.54	−3.03	433	301	49100	431
**2c**	434	305	505	2.46	−3.12	421	300	39600	453

**Table 4:** The numerical data of the cyclic voltammetry measurements.

Compound	J_SC_ [mA/cm^2^]	V_OC_ [mV]	FF [%]	PCE [%]	Dye Loading [nmol/cm^2^]	R_1_ [Ω]	R_2_ [Ω]	R_3_ [Ω]	τ [ms]
**1a**	3.34	585	72.5	1.42	0.64	20.3	3.3	145.5	4.3
**1b**	1.17	592	70.8	0.49	2.02	17.7	20.2	253.5	7.5
**1c**	1.41	519	69.2	0.51	0.51	13.0	4.9	304.0	4.3
**2a**	5.97	607	71.4	2.59	51.40	26.0	17.8	50.2	2.4
**2b**	5.82	604	72.2	2.54	35.72	24.4	4.4	68.0	5.7
**2c**	4.85	580	71.1	2.00	24.68	17.5	5.0	108.6	4.3

## Supplementary Materials

The following are available online at https://www.mdpi.com/1996-1944/13/9/2065/s1, Tables S1–S6: Table S1 Cartesian coordinates of the optimized structure of 1a in the ground state; Table S2 Cartesian coordinates of the optimized structure of 1b in the ground state; Table S3 Cartesian coordinates of the optimized structure of 1c in the ground state; Table S4 Cartesian coordinates of the optimized structure of 2a in the ground state; Table S5 Cartesian coordinates of the optimized structure of 2b in the ground state; Table S6 Cartesian coordinates of the optimized structure of 2c in the ground state.
