# Supplementary material for: 2-Thiohydantoin Moiety as a Novel Acceptor/Anchoring Group of Photosensitizers for Dye-Sensitized Solar Cells
Source: Materials (Basel). 2020 Apr 30;13(9):2065. doi: 10.3390/ma13092065 (PMC7254308; doi:10.3390/ma13092065)
Supplement: Supplementary file 1 [file materials-13-02065-s001.pdf]

*Supplementary material*

# 2–Thiohydantoin Moiety as a Novel Acceptor/Anchoring Group of Photosensitizers for Dye–Sensitized Solar Cells

**Aleksandra Bartkowiak [1], Bartosz Orwat [1,2,*], Maciej Zalas [1], Przemyslaw Ledwon [3], Ireneusz Kownacki [1,2] and Waldemar Tejchman [4]**

[1] Faculty of Chemistry, Adam Mickiewicz University in Poznań, 8 Uniwersytetu Poznańskiego St., 61–614 Poznań, Poland; aleksandra.bartkowiak@amu.edu.pl (A.B.); maciej.zalas@amu.edu.pl (M.Z.); irekk@o365.amu.edu.pl (I.K.)

[2] Center for Advanced Technology, 10 Uniwersytetu Poznańskiego St., 61–614 Poznań, Poland

[3] Faculty of Chemistry, Silesian University of Technology, 9 Marcina Strzody St., 44–100 Gliwice, Poland; przemyslaw.ledwon@polsl.pl

[4] Institute of Biology, Pedagogical University of Cracow, 2 Podchorążych St., 30–084 Kraków, Poland; waldek.tejchman@gmail.com

* Correspondence: b.orwat@amu.edu.pl

**Table S1.** Cartesian coordinates of the optimized structure of **1a** in the ground state.

| Symbol | X | Y | Z |
|---|---|---|---|
| N | −4.5533 | 0.1365 | 0.0243 |
| C | −3.1562 | −0.0715 | −0.0264 |
| C | −5.0901 | 1.4529 | −0.0837 |
| C | −5.4431 | −0.967 | 0.1744 |
| C | −6.5994 | −1.0569 | −0.6178 |
| C | −7.4797 | −2.1288 | −0.4591 |
| C | −7.2141 | −3.1331 | 0.4777 |
| C | −6.0594 | −3.0485 | 1.2631 |
| C | −5.1829 | −1.9714 | 1.1216 |
| C | −2.616 | −1.209 | −0.6552 |
| C | −1.2404 | −1.4124 | −0.6956 |
| C | −0.3402 | −0.4883 | −0.1299 |
| C | −0.8914 | 0.6505 | 0.4891 |
| C | −2.2661 | 0.8543 | 0.55 |
| C | −4.619 | 2.3382 | −1.0674 |
| C | −5.1503 | 3.6251 | −1.1679 |
| C | −6.1678 | 4.0432 | −0.3035 |
| C | −6.6447 | 3.1595 | 0.6705 |
| C | −6.1069 | 1.8764 | 0.7875 |
| C | 1.1222 | −0.7042 | −0.1837 |
| C | 1.6752 | −2.0004 | −0.1443 |
| C | 3.0488 | −2.1986 | −0.1998 |
| C | 3.9487 | −1.1121 | −0.2785 |
| C | 3.3932 | 0.1857 | −0.3341 |
| C | 2.018 | 0.3803 | −0.2895 |
| C | 5.3701 | −1.4066 | −0.3209 |
| C | 6.4517 | −0.6108 | −0.134 |
| N | 6.5615 | 0.7441 | 0.2041 |
| C | 7.8597 | 1.144 | 0.3278 |
| N | 8.6243 | 0.0365 | 0.0258 |
| C | 7.8532 | −1.0888 | −0.2563 |
| O | 8.2774 | −2.1999 | −0.5431 |
| S | 8.4168 | 2.6551 | 0.7578 |
| H | −6.8057 | −0.2864 | −1.3536 |
| H | −8.3697 | −2.1834 | −1.0795 |
| H | −7.8973 | −3.9687 | 0.5951 |
| H | −5.8443 | −3.8167 | 2.0005 |
| H | −4.2963 | −1.9046 | 1.7439 |
| H | −3.2777 | −1.9295 | −1.1237 |
| H | −0.8613 | −2.2878 | −1.214 |
| H | −0.2385 | 1.3733 | 0.969 |
| H | −2.6556 | 1.7307 | 1.0567 |
| H | −3.8404 | 2.0152 | −1.751 |
| H | −4.776 | 4.2974 | −1.9347 |
| H | −6.5839 | 5.0424 | −0.3888 |
| H | −7.4308 | 3.4718 | 1.352 |
| H | −6.4735 | 1.1989 | 1.5519 |
| H | 1.0229 | −2.8622 | −0.0476 |
| H | 3.4433 | −3.2101 | −0.1625 |
| H | 4.0251 | 1.0557 | −0.4725 |
| H | 1.6305 | 1.3906 | −0.3721 |
| H | 5.6344 | −2.4434 | −0.5161 |
| H | 5.8054 | 1.3542 | 0.4764 |

**Table S2.** Cartesian coordinates of the optimized structure of **1b** in the ground state.

| Symbol | X | Y | Z |
|---|---|---|---|
| N | −4.718 | 0.32438 | −0.1392 |
| C | −3.3238 | 0.07601 | −0.1056 |
| C | −5.5572 | −0.4128 | −1.0234 |
| C | −5.2908 | 1.31775 | 0.70616 |
| C | −6.4816 | 1.05558 | 1.40408 |
| C | −7.0481 | 2.03443 | 2.22261 |
| C | −6.431 | 3.28102 | 2.37128 |
| C | −5.2415 | 3.54169 | 1.6824 |
| C | −4.678 | 2.5739 | 0.84904 |
| C | −2.6146 | 0.1085 | 1.10898 |
| C | −1.2437 | −0.1346 | 1.13823 |
| C | −0.5254 | −0.4264 | −0.0358 |
| C | −1.2424 | −0.4544 | −1.2452 |
| C | −2.613 | −0.2072 | −1.2851 |
| C | −5.4109 | −1.8042 | −1.1517 |
| C | −6.232 | −2.5212 | −2.0237 |
| C | −7.2214 | −1.8679 | −2.7665 |
| C | −7.3757 | −0.4841 | −2.6322 |
| C | −6.5473 | 0.24216 | −1.7746 |
| C | 0.94873 | −0.6308 | −0.0286 |
| C | 1.59249 | −1.6156 | 0.80036 |
| C | 3.02803 | −1.7205 | 0.80131 |
| C | 3.80601 | −0.8656 | −0.0621 |
| C | 3.13622 | 0.03942 | −0.8797 |
| C | 1.73568 | 0.15608 | −0.858 |
| C | 5.25793 | −0.997 | −0.1248 |
| C | 6.17488 | −0.0285 | −0.3543 |
| N | 6.02021 | 1.35531 | −0.5039 |
| C | 7.2131 | 1.99575 | −0.6607 |
| N | 8.17541 | 1.00533 | −0.6557 |
| C | 7.63897 | −0.2651 | −0.465 |
| O | 8.2651 | −1.3141 | −0.4105 |
| S | 7.47028 | 3.63412 | −0.8232 |
| H | −6.9597 | 0.08659 | 1.30278 |
| H | −7.9689 | 1.81499 | 2.75556 |
| H | −6.8705 | 4.03804 | 3.01354 |
| H | −4.7547 | 4.50775 | 1.78133 |
| H | −3.7627 | 2.78828 | 0.30673 |
| H | −3.1393 | 0.32975 | 2.03227 |
| H | −0.7235 | −0.0873 | 2.09026 |
| H | −0.7202 | −0.6746 | −2.1719 |
| H | −3.1355 | −0.2346 | −2.2353 |
| H | −4.6544 | −2.3191 | −0.5683 |
| H | −6.1056 | −3.5965 | −2.1109 |
| H | −7.8629 | −2.429 | −3.4391 |
| H | −8.136 | 0.03781 | −3.2064 |
| H | −6.6649 | 1.31718 | −1.6841 |
| H | 3.69246 | 0.64573 | −1.5876 |
| H | 1.26177 | 0.89349 | −1.4983 |
| H | 5.69372 | −1.9808 | 0.02407 |
| H | 5.15856 | 1.86942 | −0.3855 |
| H | 9.16233 | 1.19908 | −0.765 |
| C | 3.6378 | −2.6701 | 1.66757 |
| C | 2.88309 | −3.5162 | 2.45336 |
| C | 1.47207 | −3.4525 | 2.41104 |
| C | 0.84738 | −2.5225 | 1.60748 |
| H | −0.2349 | −2.4836 | 1.57809 |
| H | 0.87959 | −4.1355 | 3.01236 |
| H | 3.37525 | −4.2354 | 3.1012 |
| H | 4.71868 | −2.7312 | 1.7205 |

**Table S3.** Cartesian coordinates of the optimized structure of **1c** in the ground state.

| Symbol | X | Y | Z |
|---|---|---|---|
| N | 4.97389 | 0.27563 | −0.0621 |
| C | 3.57226 | 0.06842 | 0.01617 |
| C | 5.48806 | 1.50483 | −0.5637 |
| C | 5.87137 | −0.7547 | 0.33841 |
| C | 7.00897 | −0.4492 | 1.10473 |
| C | 7.89227 | −1.46 | 1.48796 |
| C | 7.64963 | −2.7904 | 1.12954 |
| C | 6.51441 | −3.0977 | 0.37198 |
| C | 5.63557 | −2.0902 | −0.0295 |
| C | 3.00523 | −0.597 | 1.11683 |
| C | 1.62879 | −0.8089 | 1.18514 |
| C | 0.77159 | −0.3556 | 0.1704 |
| C | 1.34251 | 0.31562 | −0.922 |
| C | 2.71925 | 0.5213 | −1.0051 |
| C | 4.92974 | 2.72925 | −0.159 |
| C | 5.43146 | 3.93216 | −0.6588 |
| C | 6.50661 | 3.93678 | −1.5539 |
| C | 7.07064 | 2.71957 | −1.9504 |
| C | 6.56346 | 1.51163 | −1.4681 |
| C | −0.7057 | −0.5873 | 0.2467 |
| C | −1.283 | −1.6895 | −0.4267 |
| C | −2.7125 | −1.898 | −0.3842 |
| C | −3.5396 | −1.0053 | 0.35003 |
| C | −2.9516 | 0.06413 | 1.07721 |
| C | −1.523 | 0.28641 | 0.99893 |
| C | −4.9898 | −1.2431 | 0.35617 |
| C | −5.9752 | −0.3677 | 0.06264 |
| N | −5.9001 | 0.96876 | −0.3441 |
| C | −7.1271 | 1.50411 | −0.5983 |
| N | −8.0381 | 0.49692 | −0.3401 |
| C | −7.4306 | −0.6867 | 0.06736 |
| O | −7.9967 | −1.7311 | 0.35333 |
| S | −7.4741 | 3.04785 | −1.1166 |
| H | 7.19791 | 0.57874 | 1.39681 |
| H | 8.76641 | −1.2062 | 2.08089 |
| H | 8.33496 | −3.5751 | 1.43505 |
| H | 6.31643 | −4.1247 | 0.07832 |
| H | 4.7646 | −2.3348 | −0.6288 |
| H | 3.6442 | −0.9492 | 1.91985 |
| H | 1.21452 | −1.3255 | 2.04656 |
| H | 0.70448 | 0.67108 | −1.7264 |
| H | 3.13519 | 1.03274 | −1.8667 |
| H | 4.1041 | 2.73443 | 0.54532 |
| H | 4.98737 | 4.86933 | −0.3353 |
| H | 6.89905 | 4.87422 | −1.936 |
| H | 7.90241 | 2.70621 | −2.6491 |
| H | 6.99882 | 0.57101 | −1.7895 |
| H | −5.3426 | −2.25 | 0.56697 |
| H | −5.0491 | 1.4986 | −0.4722 |
| H | −9.0368 | 0.62113 | −0.4442 |
| C | −3.2557 | −2.9989 | −1.1225 |
| C | −2.4484 | −3.865 | −1.818 |
| C | −1.0391 | −3.6748 | −1.8381 |
| C | −0.4791 | −2.6166 | −1.1682 |
| H | 0.59412 | −2.4684 | −1.1928 |
| H | −0.409 | −4.3654 | −2.3904 |
| H | −2.8882 | −4.696 | −2.3612 |
| H | −4.3289 | −3.1498 | −1.1411 |
| C | −0.9649 | 1.39985 | 1.71114 |
| C | −1.7427 | 2.21584 | 2.49169 |
| C | −3.1349 | 1.95505 | 2.62508 |
| C | −3.7147 | 0.91409 | 1.94377 |
| H | 0.10008 | 1.58386 | 1.63122 |
| H | −1.2959 | 3.05041 | 3.02338 |
| H | −3.7404 | 2.57714 | 3.27737 |
| H | −4.7704 | 0.71508 | 2.08228 |

**Table S4.** Cartesian coordinates of the optimized structure of **2a** in the ground state.

| Symbol | X | Y | Z |
|---|---|---|---|
| N | 5.77202 | 0.12843 | 0.07926 |
| C | 4.37712 | −0.0743 | −0.0292 |
| C | 6.64967 | −0.984 | 0.23683 |
| C | 6.31568 | 1.44518 | 0.03518 |
| C | 7.46157 | 1.71361 | −0.7316 |
| C | 8.00288 | 3.00015 | −0.7616 |
| C | 7.40352 | 4.03992 | −0.043 |
| C | 6.25849 | 3.77569 | 0.71629 |
| C | 5.72094 | 2.48836 | 0.76438 |
| C | 3.59332 | 0.72783 | −0.8796 |
| C | 2.21956 | 0.53041 | −0.9751 |
| C | 1.56655 | −0.4819 | −0.2451 |
| C | 2.36239 | −1.2851 | 0.59499 |
| C | 3.73418 | −1.0848 | 0.71007 |
| C | 6.50118 | −2.1285 | −0.564 |
| C | 7.36251 | −3.2153 | −0.4041 |
| C | 8.39265 | −3.1721 | 0.54161 |
| C | 8.54812 | −2.0291 | 1.33297 |
| C | 7.68048 | −0.9447 | 1.18987 |
| C | 0.10631 | −0.6936 | −0.3524 |
| C | −0.7751 | 0.38343 | −0.5812 |
| C | −2.1487 | 0.19212 | −0.6698 |
| C | −2.7165 | −1.0939 | −0.5316 |
| C | −1.8299 | −2.1758 | −0.332 |
| C | −0.458 | −1.9806 | −0.2381 |
| C | −4.1385 | −1.3804 | −0.6013 |
| C | −5.2139 | −0.5635 | −0.4791 |
| N | −5.311 | 0.80447 | −0.2049 |
| C | −6.6035 | 1.22961 | −0.123 |
| N | −7.3926 | 0.12129 | −0.3962 |
| C | −6.6141 | −1.0247 | −0.6074 |
| O | −7.0544 | −2.1385 | −0.8569 |
| S | −7.1255 | 2.7737 | 0.22303 |
| H | 7.92484 | 0.91365 | −1.3001 |
| H | 8.88936 | 3.19089 | −1.3598 |
| H | 7.82298 | 5.04087 | −0.073 |
| H | 5.78718 | 4.57101 | 1.28671 |
| H | 4.84148 | 2.28756 | 1.36766 |
| H | 4.06629 | 1.50068 | −1.4759 |
| H | 1.65374 | 1.15144 | −1.6628 |
| H | 1.89841 | −2.0556 | 1.20333 |
| H | 4.31168 | −1.7064 | 1.38578 |
| H | 5.71303 | −2.1623 | −1.3093 |
| H | 7.23523 | −4.0925 | −1.032 |
| H | 9.06527 | −4.0163 | 0.6588 |
| H | 9.34044 | −1.9831 | 2.07464 |
| H | 7.79873 | −0.0653 | 1.81461 |
| H | −0.3816 | 1.39046 | −0.674 |
| H | −2.7701 | 1.05412 | −0.8847 |
| H | −2.2324 | −3.1816 | −0.2502 |
| H | 0.18801 | −2.8417 | −0.101 |
| H | −4.4079 | −2.4234 | −0.7502 |
| H | −4.5521 | 1.4134 | 0.06275 |
| C | −8.8351 | 0.11094 | −0.4168 |
| C | −9.4339 | −0.15 | 0.95981 |
| O | −10.775 | −0.0741 | 0.90415 |
| O | −8.8076 | −0.4029 | 1.96615 |
| H | −11.136 | −0.257 | 1.7899 |
| H | −9.1647 | −0.6809 | −1.095 |
| H | −9.2031 | 1.06711 | −0.7949 |

**Table S5.** Cartesian coordinates of the optimized structure of **2b** in the ground state.

| Symbol | X | Y | Z |
|---|---|---|---|
| N | 5.83182 | −0.3716 | 0.15224 |
| C | 4.43839 | −0.1253 | 0.07268 |
| C | 6.46166 | −1.2329 | −0.7918 |
| C | 6.60779 | 0.22692 | 1.1856 |
| C | 7.86632 | 0.78168 | 0.8986 |
| C | 8.63082 | 1.35698 | 1.9152 |
| C | 8.1489 | 1.40378 | 3.22771 |
| C | 6.89295 | 0.85825 | 3.51432 |
| C | 6.1299 | 0.2663 | 2.50641 |
| C | 3.91645 | 1.15338 | 0.33953 |
| C | 2.54633 | 1.3907 | 0.26589 |
| C | 1.6432 | 0.36942 | −0.0817 |
| C | 2.17419 | −0.9053 | −0.3466 |
| C | 3.54322 | −1.1531 | −0.2713 |
| C | 6.16828 | −1.125 | −2.1616 |
| C | 6.78403 | −1.9741 | −3.0826 |
| C | 7.71259 | −2.9303 | −2.6573 |
| C | 8.01316 | −3.0329 | −1.2949 |
| C | 7.38905 | −2.1984 | −0.3659 |
| C | 0.17238 | 0.59395 | −0.0995 |
| C | −0.4453 | 1.63051 | −0.8843 |
| C | −1.8673 | 1.83656 | −0.8023 |
| C | −2.6625 | 0.99072 | 0.05469 |
| C | −2.0268 | −0.0286 | 0.7573 |
| C | −0.6367 | −0.2212 | 0.67991 |
| C | −4.0889 | 1.24281 | 0.22503 |
| C | −5.078 | 0.34815 | 0.45753 |
| N | −5.0508 | −1.0488 | 0.50983 |
| C | −6.2855 | −1.587 | 0.71491 |
| N | −7.1573 | −0.5137 | 0.84864 |
| C | −6.4957 | 0.70975 | 0.68648 |
| O | −7.0241 | 1.81106 | 0.74491 |
| S | −6.6675 | −3.2061 | 0.80054 |
| H | 8.24164 | 0.75939 | −0.1194 |
| H | 9.60185 | 1.78099 | 1.67565 |
| H | 8.74317 | 1.85736 | 4.01502 |
| H | 6.50822 | 0.87933 | 4.53001 |
| H | 5.1627 | −0.1669 | 2.73993 |
| H | 4.58691 | 1.9617 | 0.61132 |
| H | 2.17303 | 2.38486 | 0.49289 |
| H | 1.5053 | −1.7177 | −0.6159 |
| H | 3.9212 | −2.1485 | −0.4793 |
| H | 5.45931 | −0.3765 | −2.5004 |
| H | 6.54592 | −1.8771 | −4.1381 |
| H | 8.19507 | −3.5849 | −3.3766 |
| H | 8.72755 | −3.7745 | −0.9488 |
| H | 7.61864 | −2.291 | 0.69068 |
| H | −2.5919 | −0.6444 | 1.4499 |
| H | −0.1813 | −0.9973 | 1.28704 |
| H | −4.4381 | 2.26985 | 0.16851 |
| H | −4.2558 | −1.6326 | 0.29206 |
| C | −8.5791 | −0.6175 | 1.07171 |
| C | −2.4537 | 2.85094 | −1.6086 |
| C | −1.69 | 3.6331 | −2.4501 |
| C | −0.2964 | 3.41993 | −2.5451 |
| C | 0.30522 | 2.43979 | −1.7844 |
| H | 1.37006 | 2.26628 | −1.8832 |
| H | 0.29879 | 4.01867 | −3.2279 |
| H | −2.1648 | 4.39955 | −3.0552 |
| H | −3.5269 | 3.00199 | −1.5832 |
| C | −9.3658 | −0.7022 | −0.2306 |
| O | −8.8955 | −0.6321 | −1.3455 |
| O | −10.676 | −0.8567 | 0.02799 |
| H | −8.9093 | 0.26825 | 1.62134 |
| H | −8.7901 | −1.5009 | 1.67823 |
| H | −11.159 | −0.8972 | −0.8167 |

**Table S6.** Cartesian coordinates of the optimized structure of **2c** in the ground state.

| Symbol | X | Y | Z |
|---|---|---|---|
| N | 5.93282 | 0.3054 | −0.1832 |
| C | 4.53922 | 0.07681 | −0.0535 |
| C | 6.40962 | 1.51264 | −0.7691 |
| C | 6.86356 | −0.675 | 0.26517 |
| C | 8.0049 | −0.2932 | 0.99032 |
| C | 8.92054 | −1.2552 | 1.42066 |
| C | 8.70688 | −2.6111 | 1.15076 |
| C | 7.56814 | −2.9941 | 0.43386 |
| C | 6.65692 | −2.0363 | −0.0145 |
| C | 4.01689 | −0.5476 | 1.09277 |
| C | 2.64734 | −0.7779 | 1.21312 |
| C | 1.75103 | −0.3866 | 0.20582 |
| C | 2.27812 | 0.24191 | −0.9335 |
| C | 3.64743 | 0.46766 | −1.0677 |
| C | 5.84103 | 2.74912 | −0.4195 |
| C | 6.30853 | 3.92903 | −1.0008 |
| C | 7.35926 | 3.8989 | −1.924 |
| C | 7.93362 | 2.67003 | −2.2659 |
| C | 7.46007 | 1.48406 | −1.7018 |
| C | 0.28174 | −0.6355 | 0.34042 |
| C | −0.3249 | −1.6953 | −0.3755 |
| C | −1.7497 | −1.9155 | −0.2767 |
| C | −2.5411 | −1.0871 | 0.56522 |
| C | −1.9208 | −0.0669 | 1.33484 |
| C | −0.501 | 0.17844 | 1.19191 |
| C | −3.9851 | −1.3458 | 0.63437 |
| C | −4.9999 | −0.4689 | 0.47238 |
| N | −4.9754 | 0.89612 | 0.17772 |
| C | −6.2232 | 1.42257 | 0.03359 |
| N | −7.1124 | 0.37693 | 0.25909 |
| C | −6.4402 | −0.8212 | 0.52542 |
| O | −6.9739 | −1.8971 | 0.75091 |
| S | −6.6079 | 3.00099 | −0.3285 |
| H | 8.17166 | 0.75566 | 1.21346 |
| H | 9.79716 | −0.9424 | 1.98083 |
| H | 9.41711 | −3.3577 | 1.49289 |
| H | 7.39244 | −4.0422 | 0.20904 |
| H | 5.78311 | −2.3399 | −0.582 |
| H | 4.68538 | −0.8525 | 1.8909 |
| H | 2.26919 | −1.2613 | 2.10966 |
| H | 1.61021 | 0.54979 | −1.7333 |
| H | 4.02761 | 0.94741 | −1.9633 |
| H | 5.0348 | 2.78181 | 0.30622 |
| H | 5.85715 | 4.87621 | −0.7193 |
| H | 7.72504 | 4.81888 | −2.3697 |
| H | 8.74668 | 2.6297 | −2.9852 |
| H | 7.90283 | 0.53331 | −1.9806 |
| H | −4.3119 | −2.3733 | 0.77605 |
| H | −4.1443 | 1.45586 | 0.04661 |
| C | −8.5499 | 0.47498 | 0.1798 |
| C | −2.327 | −2.9628 | −1.0651 |
| C | −1.5533 | −3.776 | −1.8561 |
| C | −0.1461 | −3.5838 | −1.9239 |
| C | 0.44413 | −2.5715 | −1.2102 |
| H | 1.5157 | −2.423 | −1.2707 |
| H | 0.45874 | −4.2361 | −2.5465 |
| H | −2.0186 | −4.5672 | −2.4364 |
| H | −3.4 | −3.1141 | −1.046 |
| C | 0.08263 | 1.25594 | 1.93813 |
| C | −0.6571 | 2.00624 | 2.81581 |
| C | −2.034 | 1.71137 | 3.01727 |
| C | −2.6406 | 0.70911 | 2.30213 |
| H | 1.13679 | 1.46737 | 1.8041 |
| H | −0.1905 | 2.81471 | 3.37034 |
| H | −2.6064 | 2.27764 | 3.74586 |
| H | −3.6833 | 0.48315 | 2.49 |
| C | −9.069 | 0.23761 | −1.2331 |
| O | −10.402 | 0.40649 | −1.2726 |
| O | −8.3928 | −0.0698 | −2.1909 |
| H | −10.712 | 0.23883 | −2.1804 |
| H | −8.9851 | −0.2781 | 0.8423 |
| H | −8.8688 | 1.46225 | 0.52063 |
